# Probing the internal micromechanical properties of *Pseudomonas aeruginosa* biofilms by Brillouin imaging

**DOI:** 10.1038/s41522-017-0028-z

**Published:** 2017-09-08

**Authors:** A. Karampatzakis, C. Z. Song, L. P. Allsopp, A. Filloux, S. A. Rice, Y. Cohen, T. Wohland, P. Török

**Affiliations:** 1 Centre for BioImaging Sciences, National University of Singapore, Singapore, 117557 Singapore; 2 NUS Graduate School for Integrative Sciences and Engineering, National University of Singapore, Singapore, 117557 Singapore; 3 Blackett Laboratory, Department of Physics, Imperial College London, Prince Consort Road, London, SW7 2BZ United Kingdom; 4 Imperial College London, Department of Life Sciences, MRC Centre for Molecular Bacteriology and Infection, South Kensington Campus, Flowers Building, SW7 2AZ London, United Kingdom; 5 Singapore Centre for Environmental Life Sciences Engineering and the School of Biological Sciences, Nanyang Technological University Singapore, Singapore, 637551 Singapore; 6 Department of Biological Sciences and Department of Chemistry, National University of Singapore, Singapore, 117346 Singapore

## Abstract

Biofilms are organised aggregates of bacteria that adhere to each other or surfaces. The matrix of extracellular polymeric substances that holds the cells together provides the mechanical stability of the biofilm. In this study, we have applied Brillouin microscopy, a technique that is capable of measuring mechanical properties of specimens on a micrometre scale based on the shift in frequency of light incident upon a sample due to thermal fluctuations, to investigate the micromechanical properties of an active, live *Pseudomonas aeruginosa* biofilm. Using this non-contact and label-free technique, we have extracted information about the internal stiffness of biofilms under continuous flow. No correlation with colony size was found when comparing the averages of Brillouin shifts of two-dimensional cross-sections of randomly selected colonies. However, when focusing on single colonies, we observed two distinct spatial patterns: in smaller colonies, stiffness increased towards their interior, indicating a more compact structure of the centre of the colony, whereas, larger (over 45 μm) colonies were found to have less stiff interiors.

## Biofilm structure: Shining a light on stiffness

A specialized microscopy technique can monitor biofilm stiffness in a non-destructive manner, yielding insights into biofilm structure and development. The technique, called Brillouin imaging, uses changes in the frequency of light interacting with a substance to reveal fine detail about the material’s mechanical properties. Peter Török and colleagues at Imperial College London, with co-workers in Singapore, used Brillouin imaging to study biofilms of *Pseudomonas aeruginosa* bacteria at different stages in their life cycle. In young colonies, stiffness increased towards the interior of the biofilm, while mature colonies had less stiff interiors. The older biofilms may therefore have hollow interiors or may have been moving towards a phase of bacterial dispersal from the biofilm state. This non-disruptive method to study mechanical variations within and between living biofilms may help efforts to combat biofilms in clinical, environmental and industrial situations.

## Introduction

Biofilms are developed when extracellular polymeric substances (EPS) are secreted from adherent bacterial cells to form a matrix that encloses bacterial cells.^1^ They readily adhere to biological or non-biological surfaces, are highly dynamic and heterogeneous,^2–4^ yet have a distinct lifecycle. The proliferation of biofilms in many environmental settings may be the result of selective processes resulting in many ecological advantages of the biofilm mode of life, such as their inherent resistance to antibiotics^5^ and difficulty in removing them from a contaminated surface.^6^ Biofilms are everywhere and have a major impact on numerous domains of society: the majority of bacterial infections are biofilm-related,^7^ they are responsible for persistent contamination in the food and dairy industry^8^ and fouling of water systems.^9^

The EPS accounts for the largest part of the dry mass of biofilms (up to 90%)^10^ and is responsible for the formation and maintenance of biofilms and their three-dimensional architecture. The matrix is a cross-linked network of polymers and has presumably multiple roles in the life of biofilms. Amongst these roles, the matrix acts as a protective barrier, facilitates adhesion on surfaces, acts as a nutrient source and retains water in close proximity to the bacterial cells.^1^ Remarkably, it has been found that the matrix changes its properties in dynamic mode throughout the lifecycle of the biofilms.^11^ For example, in *Pseudomonas aeruginosa* biofilms, Psl, an exopolysaccharide, contributes to the stiffening and formation of the matrix.^12^ As biofilms colonies grow, the localisation of Psl changes, leading to softening of the colony centres and the formation of hollow colonies.^13, 14^ Being linked with such a large number of functionalities, it is not surprising that the mechanical properties of the EPS have been a subject of interest for many years. A number of techniques at different scales have been employed, such as atomic force microscopy (AFM),^15, 16^ magnetic tweezers,^17^ small-scale bulk rheometry,^6^ microbeads AFM,^18^ single particle tracking,^19^ video particle tracking^12^ and others.^20^ All the aforementioned techniques have some limitations: they either require direct interactions between the sample and a probe; they are able to measure only in discreet positions and only near surfaces; or they are suitable only for bulk measurements.

In the present study, we applied confocal Brillouin microscopy^21^ to study the mechanical properties of biofilms formed by *P. aeruginosa*, an opportunistic bacterial pathogen commonly used as a model organism for biofilm formation. The term confocal Brillouin microscope (CBM) refers to a conventional confocal microscope equipped with an ultra-high resolution spectrometer as detector. One parameter extracted from CBM measurements is the frequency shift of the inelastically scattered light that occurs due to the energy transfer between the incident photons and the thermal acoustic waves, termed acoustic phonons, that are propagating within the sample. Stiffness information can be inferred from this frequency shift,^22–24^ which is typically in the range of 5–20 GHz. The Brillouin shift is measured at each position of the sample, as it is scanned with respect to the focused illumination produced by a single frequency laser.

The biggest challenge in Brillouin microscopy is detecting and isolating the Brillouin signal. It is usually orders of magnitude weaker than the elastically scattered light and the frequency shift is below the spectral resolution limit of conventional spectrometers. Historically, in the first applications, high-finesse scanning Fabry–Perot interferometers or angle-dispersive etalons were used, however, these devices are too slow to provide a satisfactory data acquisition speeds for biological samples.^25^ Technological advancements have brought about virtually imaged phase arrays (VIPAs),^26^ which were subsequently introduced in Brillouin microscopy.^22^ This device permits faster data acquisition speeds as compared to conventional Fabry–Perot etalons. Because the signal strength of the Brillouin scattered light is small compared to the elastic signal, a single VIPA often does not allow clear separation of the Brillouin peak from the background. It is well known in spectroscopy that applying a number of sequential spectrometers improves the suppression of the non-specific background that in turn permits clear differentiation of the Brillouin peak. For example, cascading two and three VIPAs in a cross-axis configuration has shown a 55 dB and 80 dB signal-to-noise improvement, respectively.^27^ Other methods that were used for the non-specific background suppression are molecular absorption cells (up to 50 dB suppression),^28^ Michelson interferometer,^29^ multi-pass Fabry-Perot interferometer,^30^ tunable etalon-based notch filters^31^ and common path interferometric filtering.^24^ These improvements, together with background studies that showed the applicability of high numerical aperture objective lenses,^32^ have allowed Brillouin spectroscopy to find a number of applications in the life sciences and medicine, such as for in situ and in vivo biomechanical imaging of the eye tissues,^22, 24, 27, 33^ quantification of plaque stiffness in arteries^23^ and in single cell studies.^34, 35^

Here we applied confocal Brillouin microscopy to study the stiffness of *P. aeruginosa* biofilms in a non-destructive fashion, in real time. We used a custom built CBM with two, cross-axis VIPA spectrometers and common path interferometric filter to achieve a high non-specific background suppression (95 dB overall) suitable for imaging thick, turbid samples. We measured the Brillouin frequency shift within living biofilms as they grow inside a flow cell. We investigated the relationship of frequency shift with a number of factors, such as the colony size, depth of imaging and flow speed to establish micromechanical properties of biofilms. This study demonstrates the utility of Brillouin microscopy for investigating, at high resolution, the mechanical properties of the interior parts of living, growing biofilm colonies.

## Results

### Characterisation of the stiffness of *P. aeruginosa* biofilm

**Fig. 1:**
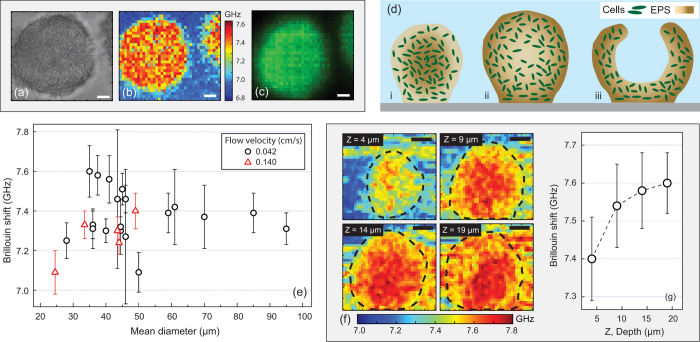
Characterisation of the stiffness of *P. aeruginosa* biofilms. Typical **a** widefield, **b** Brillouin, and **c** fluorescence images of a single colony taken 60 h post inoculation at a depth of 24 μm inside a 42-μm thick biofilm. **d** Schematic model defining the various stages of a *P. aeruginosa* biofilm life cycle. Compact colony (i), larger colony with softer centre (ii), and hollow colony (iii). **e** Brillouin shift in colonies of various sizes. Data points denote the means, and error bars the standard deviations from all pixels within the ROIs enclosing the colony. ROIs drawn by visual inspection of the corresponding widefield images. Biofilms grown under constant flow velocities of either 0.042 or 0.14 cm/s (circles and triangles, respectively). **f** Brillouin image cross sections at different depths inside a single colony (thickness 32 μm, taken 80 h post insoculation). **g** Mean values and standard deviation of the Brillouin shift at different depths, measured within the ROIs marked in f. Data points connected by lines to aid visualisation. Scale bars: 10 μm

We used Brillouin microscopy together with fluorescence imaging to image a gfp-tagged *P. aeruginosa* PAO1 biofilms. A total of 24 different biofilm colonies of various sizes, average diameters ranging from 25 to 95 μm and thicknesses from 16 to 55 μm, were imaged at various depths and at different time points after inoculation. Here, biofilm colonies are defined as the visually observable convex structures of bacteria aggregates that rise above the flat surrounding, characterised by a circumference and height. Video S.1 (Supplementary Material) shows a *z*-stack of a single colony imaged with the aforementioned modalities. Altogether we observed gradients of stiffness, signified by an increase in Brillouin shift with respect to the surrounding background. Two different patterns were identified: 21 out of the 24 imaged colonies were found to have increasing stiffness and biomass towards their centres, as shown by the Brillouin and fluorescence images, respectively (Fig. 1a–c, example). In the remaining three colonies, the interior was less stiff than the periphery.

The mean and standard deviations of the Brillouin shifts, measured within regions of interest (ROI) enclosing the whole colony, are plotted against the horizontal diameter of the colony in Fig. 1e. No correlation between colony size and average stiffness is detected. It should be noted that the error bars, which denote standard deviations, may well be the result of the heterogeneity of the sample enclosed by the ROI.

### Brillouin shift measured at increasing depths

*P. aeruginosa* biofilms have been previously shown to form colonies that expand into pillar-like and mushroom-shaped colonies in flow cells like the one used in our study.^36^ A *z*-stack of Brillouin images was taken in a colony of thickness 32 μm. We define *Z* = 0 at the vertex of each colony and therefore *z* increases with increasing depth towards the substrate. The stiffness was observed to increase towards the centre of each cross-section at the different depths (Fig. 1f), as well as overall with increasing depth (Fig. 1g).

**Fig. 2:**
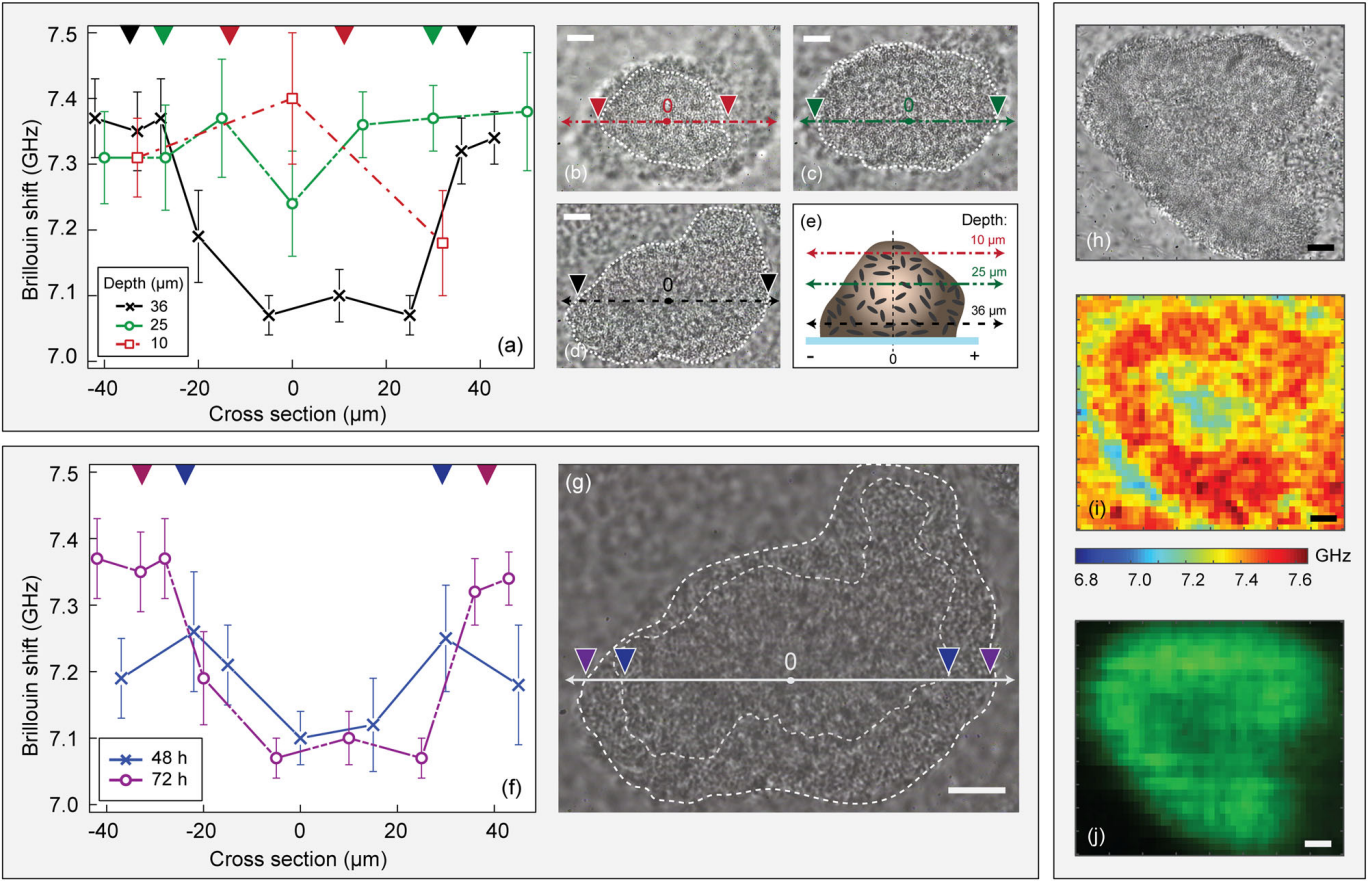
Brillouin imaging in large biofilm colonies revealing areas of decreased stiffness in their centres. **a** Brillouin shift measured along a cross-section of a single colony (thickness 38 μm, taken at 72 h post inoculation), at three defined depths. Data points and error bars represent the mean and standard deviations from ten technical repeats at each point. The colour of the triangles on the top border denotes the colony boundaries at each depth corresponding to panels b–d. **b**–**d** Widefield images of the same colony at different depths of 10, 25 and 36 μm, respectively. The white dashed lines define the in-focus area, which was visually defined to represent the boundaries of the colony at each depth. **e** Schematic illustrating the imaged cross-sections. Darker colour indicates increasing stiffness. **f** Brillouin shifts measured along the cross-section of a single colony at 48 and 72 h post inoculation. Data points and error bars represent the mean and standard deviations from 10 technical repeats taken at each point. The color of triangles on the top border denote the colony boundaries at the two time points and correspond to panel g. **g** Widefield image of the same colony, taken 72 h post inoculation The white dashed lines define the in-focus area, which was visually defined to represent the boundaries of the colony at each time point. **h** Widefield, **i** Brillouin, and **j** fluorescence images of a different hollow colony imaged 100 h post inoculation taken at a depth of 15 μm inside a 35-μm thick biofilm. Scale bars: 10 μm

To investigate the internal stiffness profiles in larger colonies, we had to modify the imaging protocol to decrease the acquisition time. Here, instead of acquiring complete Brillouin images at different depths, we performed repeated measurements at points along a cross section running through the middle of the colony, at various depths (Fig. 2a–e). Widefield images of the cross sections are shown in Fig. 2b–d. Figure 2a shows a decrease in stiffness towards the middle of the cross section when imaging at the depths of 36 and 25 μm (as indicated by the black and green lines), while there was an increase in stiffness towards the centre of the cross section when imaging close to the top of the colony (depth = 10 μm, red line). Such a profile indicates the existence of a shell of higher stiffness that includes a softer core, or a void (Fig. 2e).

### Temporal changes of stiffness profile

Given the dynamic heterogeneous nature of biofilms,^3^ we found no correlation between the average Brillouin shifts of colonies and the time of imaging post inoculation (Supplementary Fig. S5). However, in order to observe changes in one defined colony over time, we imaged a single colony at 48 h post inoculation, fixed the position of the stage and repeated measurements of the same colony after 24 h.

The Brillouin shifts were measured both days along a line cross section at fixed depth at locations inside, near the border and outside of the colony, and are shown on Fig. 2f. It can be seen that the difference in stiffness between the periphery and the interior of the biofilm became more pronounced at 72 h. Between these two measurements, the colony had grown in size, from 55 to 74 μm in diameter and from 33 to 38 μm in thickness (Fig. 2g).

Lastly, Fig. 2h, i, j shows images of a large (85 μm mean diameter) different colony, taken 100 h post inoculation. The profile indicates a hollow colony, possibly correlating to the model shown in Fig. 1d(iii), and closely resembles images of hollow colonies shown in previous studies using similar flow cells.^37^

### Effect of flow velocity

The flow rate was kept constant throughout the whole experiment. Most of the imaged colonies (19 out of 24) were studied under 0.042 cm/s, while the rest under 0.14 cm/s. We observed that colonies exposed to the higher flow velocity were generally smaller in size as well as flatter. However, for these two distinct conditions that were tested, no effect of the flow velocity on the average stiffness of the colonies was detected (Fig. 1e).

## Discussion

In this study, we investigated the effect of several factors on the stiffness of biofilms. All measurements were performed on living samples. This was achieved using Brillouin microscopy, a novel, non-contact method, to probe in vivo the stiffness of active *P. aeruginosa* biofilms growing in a defined flow cell.^38^ We have clearly observed distinct patterns in the internal stiffness of colonies, indicative of the highly dynamic and heterogeneous nature of biofilms.^3^

For most (~88%) of the examined biofilm colonies, the centres of the colony were stiffer than their peripheries, corresponding to a model of growth on the outer edges of the colonies, with a denser core.^39^ Contrastingly, we found some colonies in which the stiffness decreased towards their centres and within such colonies, rotating bacteria were frequently observed (Video S.2). We suggest that these colonies might be in transition to the dispersal phase as it was shown that a softening of the inner parts of the colonies is expected before hollows are formed.^39–41^ All of the colonies with interiors softer than their peripheries were at least 45 μm in size (mean diameter). However, this type of colonies were often found alongside the other type where increasing stiffness towards the centre was observed, reflecting the complex nature of biofilms.^2, 3, 42^

We have shown that the stiffness of compact colonies increased with depth (Fig. 1f, g). On the other hand, no correlation was found between the size of the colony and the average stiffness when comparing two-dimensional cross-sections of different colonies (Fig. 1e). This observation is different from the findings of a previous study^16^ that targeted the stiffness of the surface of colonies by utilising AFM, while our study focused on the interior stiffness of the colonies. Lastly, in agreement with a previous study, which reports that “the Young’s modulus of the superficial layer of colonies was independent of flow velocity” (ref. 16), we did not detect an effect on stiffness between the two flow velocities that were tested.

All images shown in this study were taken between 36 and 120 h post inoculation of the flow cell. Earlier than that, colonies were too thin to image, as the elastic background scatter proximal to the coverslip is too strong to suppress. When we imaged the same colony at 48 and 72 h after inoculation, we found that a profile was established where the interior of the colony becomes less stiff than its periphery (Fig. 2f). This observation can be interpreted according to the model shown in Fig. 1d or to the dynamic heterogeneity of biofilms.^2, 3, 42^

It is worth noting that, as shown in Fig. 2a, f, the Brillouin shift measured closely outside of the visible boundaries of the colony was higher than that of the minimal medium around the colonies (~7.05 GHz). This observation can be attributed to rotating bacteria (as observed in previous studies^41^) which can move into the focal volume during the acquisition time of 900 ms, or could be due to the flocculent nature of the surfaces of the colony.

The applicability of Brillouin microscopy on biofilms was first assessed by imaging cross-linked alginate acid–calcium chloride (CaCl_2_) hydrogel beads (Supplementary Fig. S4), which are commonly used as model synthetic matrices.^43^ It should be noted that biofilms are much more complex systems than hydrogel beads, hence, the quantitative results obtained from the measurements in the beads are not—and should not be—used as a calibration look-up table to infer the composition of biofilms. However, the increased degree of heterogeneity of biological samples does not invalidate the results since only the probe volume (<1 µm^3^) is considered uniform and isotropic, and not the whole sample.

Some care needs to be taken when interpreting the data; Brillouin microscopy measures the frequency shift, *U*_b_, due to Brillouin scattering at each position of the sample, as it is being scanned with respect to the focused light distribution produced by an objective lens. Equation (1) shows that the shift is linearly proportional to the average hypersound velocity, *V*, within that volume and the refractive index, *n*. In turn, *V* is proportional to the ratio of the Brillouin modulus, *M*, a measure of stiffness, and inversely proportional to *ρ*, the material density. Considering the above, it is evident that pixel-to-pixel variations of the measured *U*_b_ could arise from changes in either (or from any combination of) *n, M* and *ρ*. First, let us address possible variations of *n*. Authors in a previous study^44^ have made the assumption that “since the biofilm has a high water content, *n* can be assumed equal to 1.333”. In a later study, this was experimentally confirmed as the refractive index of *P. aeruginosa* was measured *n* = 1.33 ± 0.17 (by optical density experiments^45^) and *n* = 1.348 ± 0.013 using a refractometer.^45^ Therefore, possible variations in the refractive index (if any) would be within the detection limits of our Brillouin microscope, as noted in a previous study.^29^

Next, we discuss the dependence of Brillouin shift on local spatial variations of density. To the best of our knowledge, no study on the density of *P. aeruginosa* biofilms with characteristics comparable to those of our study exists. However, on the basis of results published by others, using terminal velocity of particles, the biofilm density was calculated 1.14 g/cm^3^ in biofilms of wastewater.^46^ In a later study, it was calculated that the wet density of biofilms of mixed filamentous bacteria and bacillus ranges between 1 and 1.1 g/cm^3^ corresponding to dry densities between 0 and 0.05 g/cm^3^, respectively.^47^ If we were to assume that changes in *U*_b_ result from the variations in *ρ* alone, it would follow that *U*_b_ in denser materials (e.g., biofilms) would be smaller to that of water (*U*_b water_ = 7.04 GHz), which is not the case in our measurements. Therefore, the observed values of *U*_b_ are proportional to the Brillouin modulus *M*, which hence represent a measure of stiffness, even though it does not seem to be possible to assign an absolute metric to *M* without the exact knowledge of the local material density.

To further support our argument, we only found a single study that attempts to make spatial differentiation of density within biofilms in which the authors measured 1.001–1.003 g/cm^3^ at the top region of biofilms and 1.01–1.02 g/cm^3^ at the deeper regions.^48^ That is a relative change of ~1.3% that occurs over the spatial extent of several hundred of micrometres to a few millimetres (their biofilms were much thicker than those in our study). For reference, the *z*-stack shown in our Fig. 1f, g shows a 2.6% increase in Brillouin shift over only ~20 μm of depth gain, which, because it is twice the range of what was observed in ref. 48, is unlikely to be due to density differences.

Therefore, as we have shown that the observed changes in *U*_b_ cannot be explained by changes in density, we can infer stiffness directly from the Brillouin shift and not by calculating the hypersound velocity (which would require knowledge of *n* at each pixel) or the Brillouin modulus (which would require knowledge of both *n* and *ρ*, at each pixel). This assumption is similar to other published studies.^22, 24, 29, 35^ It should be noted, however, that we cannot differentiate with certainty whether the pixel-to-pixel differences of Brillouin signal are due to absence of bacterial cells or due to different properties of the EPS matrix itself.

Limitations of this study include the restriction of measurements to colonies that had distinct boundaries, visible by widefield microscopy, whereas the flat, undifferentiated portions of the biofilms or the biofilm substratum were not investigated. Additionally, given the dynamic nature of biofilm development, it remains unclear whether some of the larger colonies imaged are single colonies, or aggregates of merged smaller colonies. Lastly, the ROIs used to calculate the average Brillouin shifts were defined manually by visual inspection of the widefield images. Nonetheless, our classification is consistent.

In conclusion, we have demonstrated that Brillouin microscopy is suitable to probe the internal stiffness of bacterial biofilm colonies at micrometre scale and clearly identify relative changes in stiffness. *P. aeruginosa* biofilm colonies growing in a flow cell were measured in vivo without disrupting the flow conditions or damaging the samples, meaning that biofilms can be measured to obtain dynamic information on the change in stiffness.

Brillouin imaging does not require labelling and has significant potential for further studies of mechanical properties of complex biofilms in natural, industrial or medical settings. Given the emerging complexity of the biofilm mode of life, further optimisation of Brillouin microscopy along with stringent experimental procedures are required to unravel the mechanisms driving biofilms. Additionally, in future studies, Brillouin microscopy could work in tandem with other spectroscopic techniques that provide information on local diffusivity, porosity and viscosity, towards the better understanding of the role of mechanical properties in biofilm processes, such signalling and transport of nutrients within biofilm communities.

## Methods

### Brillouin spectroscopy

Spontaneous Brillouin scattering (or simply Brillouin scattering) refers to the inelastic scattering of light by thermal vibrations present in the sample (termed acoustic phonons) that shifts the frequency of the light by an amount equal to that of the ultrasonic wave.^33^ In isotropic materials the spectral shift (Brillouin frequency shift, *U*_B_) is proportional to the velocity of sound, *V*, in the sample according to:

1 $${U_{\rm{B}}} = \frac{{2n}}{\lambda }V\sin (\theta /2),$$

where *n* the refractive index, *λ* the optical wavelength of illumination and *θ* the scattering angle, which is set by the design of the experiment.

In isotropic and homogeneous materials the speed of sound *V* can be related to the mechanical properties of the sample via $V = \sqrt {M/\rho } ,$where *M* is the Brillouin modulus and *ρ* is the density. It should be noted, however, that a conversion of the measured frequency shift to absolute values that are traditionally used in biomechanical measurements, as for example Young’s modulus (*E*), is not currently possible for a number of reasons: firstly, Eq. (1) holds in general for isotropic materials, while, in anisotropic media, there exists a separate *M* in each direction corresponding to direction-dependent hypersound velocities, i.e., *M* is a tensor. Secondly, the refractive index and material density are not necessarily known in a point-wise manner, and lastly, the relationship between macromechanical and micromechanical quantities is, in most cases, complicated and very much dependent upon the medium being investigated. Despite the lack of a theoretical model or proper understanding, some authors attempted to use an empirical relationship between the Brillouin modulus and the quasi-static Young’s modulus although it is doubtful how this could be justified given the frequency dependent nature of Young’s modulus. In order to avoid this problem, this study uses Brillouin shift values to measure elastic properties of samples and materials with a larger Brillouin frequency shift will be referred to as ‘stiffer’ for simplicity, similarly to other existing studies.^23, 24, 35^

### Confocal microscope for Brillouin and fluorescence imaging

The Brillouin fluorescence confocal microscope (Supplementary Fig. S1a) was constructed using a commercial Olympus IX-71 microscope platform. The laser source for Brillouin imaging is a CW diode-pumped solid state laser (Cobolt 05-01 Jive) operating at 561 nm with a maximum power of 300 mW. The fluorescence excitation laser is a diode laser (Cobolt 06-01 488) operating at 488 nm with maximum power of 60 mW.

Two laser beams were delivered to the microscope using optical fibres (kineFLEX), combined by a long pass dichroic mirror (DM1) (Chroma T550lpxr) and then expanded by 6x beam expanders to fill the entrance pupil of the objective. The beams were split by a beam splitter (BS) (Thorlabs BS019) with a power ratio of 30:70 (R:T). The transmitted beams were reflected by a mirror (M) to a 10X microscope objective, which focuses the beams into the water cuvette for calibration purposes. The reflected beams were directed to the oil–immersion microscope objective lens (Nikon UPLFLN / 100X, NA = 1.3) for the Brillouin and fluorescence imaging.

Light collected from the sample was spectrally separated the dichroic mirror DM2 (Chroma ZT488/561TPC), which transmits the Brillouin and Rayleigh signals and reflects the fluorescence signal. Seventy percent of the Brillouin and Rayleigh signals were transmitted by the BS and coupled into the fibre to be delivered to the common path interferometric filter. The fluorescence signal was further filtered by an emission filter (Chroma ET525/50). A motorised pinhole (Thorlabs MPH16) was used to control the amount of fluorescence signal from the focal plane and to couple the signal to a multi-mode fibre that delivers the signal to the detector. A photon counting detector (Hamamatsu H10682-110) together with a data acquisition unit (NI USB-6351) was used to measure the fluorescence signal strength.

### Common path interferometer

A common path interferometer was used to suppress the elastically scattered light.^24^ Light delivered from the microscope through the fibre was collimated to achieve a beam diameter of 4 mm. The glass slab was aligned so that half of the beam was transmitted through the slab while the other half propagated through the glass (Supplementary Fig. S1b). The optical path difference (*δ*) between the two parts of the beam that depends on the angle between the prism and the beam (*θ*) is given by:

2 $$\delta = d(n - 1)\left( {1 + \frac{{{\theta ^2}}}{{2n}}} \right).$$

Where *d* is the length of the glass slab and *n* is its refractive index. Eq. (2) is valid for small values of *θ*.

Destructive interference occurs when the two halves of the beam overlap upon and couple into a single-mode fibre. The exact wavelength at which destructive interference occurs can be adjusted by titling the prism to vary the angle *θ*. The transmission function is a cosine function with a free spectral range (FSR) of *c/δ*, where *c* is the speed of light in vacuum. A *d* = 30 mm long BK7 glass prism (*n* = 1.51) was used in our experiment to achieve a FSR of 20 GHz. A maximum extinction of 40 dB can be achieved with our 1 MHz linewidth laser source, which was confirmed experimentally.

### VIPA spectrometer

A two stage, crossed-axis VIPA spectrometer (Supplementary Fig. S1c) was used to extract the Brillouin frequency shifts in our experiments.^27, 35^ Both VIPA 1 and VIPA 2 were custom-built by LightMachinery Inc. with a FSR of 39.5 ± 0.5 GHz.

Light from the interferometric filter output fibre was collimated to produce 0.35 mm beam waist and focused by a 100 mm cylindrical lens (C1) onto the first VIPA entrance window. Another 100 mm cylindrical lens (C2) was used to form the spectrum at its focal plane. A mask (Mask 1) is used to shape the spectrum. A 100 mm spherical lens (S1) was used to focus the light further onto the second VIPA whose axis was orthogonal to the first VIPA. A second 100 mm spherical lens (S2) forms the spectrum at its focal plane where a second mask (Mask 2) was used to shape the spectrum. An achromatic doublet pair is used to image the spectrum onto a sCMOS camera (Andor Neo 5.5 sCMOS). A typical spectrum recorded from a biofilm sample using the interferometric filter with 600 ms acquisition time is shown (Supplementary Fig. S1d).

### Data acquisition and processing

Scanning and data acquisition was controlled by in-house developed Labview (National Instruments, Austin TX, USA) software and data were analysed using Matlab (The MathWorks Inc., Natick, MA, USA). Typical exposure times varied between 500–900 ms for the Brillouin and 10–100 ms for the fluorescence imaging. A 50-μm pinhole was used for the fluorescence imaging. The microscope was calibrated using a water sample before the start of every experiment to establish the slope of the dispersion axis, and the frequency separation per pixel (*FxP*, see Supplementary Material). Consequently, as the sample was scanned, both the Stokes and anti-Stokes Brillouin peaks were captured. Their positions, determined by fitting a Lorentzian function on the spectrum, were used to calculate the frequency shift at every point of the sample, as ${U_{\rm{B}}} = \frac{{{\rm{FSR}} - FxP \times \left( {{B_2} - {B_1}} \right)}}{2}$, where *B*_1_ and *B*_2_ are the spatial position of the Stokes and anti-Stokes Brillouin peaks along the dispersion axis (Supplementary Fig. S3).

### Bacterial strains and growth conditions

gfp-tagged *P. aeruginosa* strain PAO1 was prepared according to (refs. 49, 50).

### Flow cell biofilm assays

Biofilm formation under continuous flow was performed as described in refs. 34, 38. A commercial flow cell system consisting of a 2 L medium bottle, FEP tubing (30 mm long, 2.1 mm ID and 2.3 mm OD) (Masterflex, Germany), a bubble trap (Technical University of Denmark), a flow cell (made by attaching—using Silicone glue (3 M Super Silicone Sealant Clear)—a 50 × 24 mm glass coverslip on a pre-fabricated polycarbonate base on which three channels were milled, each 1 mm deep × 4 mm wide × 40 mm long, shown in Supplementary Fig. S6a) (Technical University of Denmark) and a 2 L waste bottle was assembled and used in all experiments. Technical drawings and a protocol for constructing this flow cell can be found at ref. 38 A six roller peristaltic pump (Langer Instruments, USA) was used to pump the medium through the biofilm flow cell at a flow rate of 6 or 20 mL/h, giving rise to flow speeds of 0.042 and 0.14 cm/s, respectively. A stage was specifically manufactured for the flow cell to be placed under the microscope (Supplementary Fig. S6b–d). The FEP tubings were autoclaved and primed with a minimal medium ([15 mM (NH_4_)_2_SO_4_, 34 mM Na_2_HPO_4_, 22 mM KH_2_PO_4_, 58 mM NaCl, 1 mM MgCl_2_, 0.1 mM CaCl_2_ and 0.01 mM FeCl_3_], supplemented with 2 g/L of glucose plus 2 g/L of casamino acids) before the start of every experiment. gfp-tagged *P. aeruginosa* were grown overnight in 40 g/L LB medium (Difco) in a shaking incubator at 37^o^C and 200 rpm. The culture was diluted to OD_600_ ~2.0 in minimal medium, and 300 μL was injected into the FEP tubing near the entry port of each of the channels of the flow cell. The inoculum was allowed to attach in the flow cell for 1 h without flow, at room temperature. The flow of the minimal medium was then resumed and kept constant throughout the experiment.

### Hydrogel bead preparation

Sodium alginate (Acros Organics, U.K.) solutions (concentrations of 1 and 3%) in distilled water (dH_2_O) were prepared and autoclaved. Droplets of 10–20 μL of the prepared sodium alginate solution were gently dropped into the wells of a six-well plate (Nunclon Flat, Thermoscientific containing filter-sterilised CaCl_2_ (100 mM and 400 mM), using a pipette. The drops were left to gelate and settle for 30 min before they were washed and the CaCl_2_ was replaced with sterile dH_2_O for storage and later use.

### Sample size and repeatability information

In total, 24 different biofilm colonies, selected randomly by visual inspection, were imaged. Only colonies that had distinct boundaries, visible by widefield microscopy, were imaged at random time points that ranged between 36 and 120 h post inoculation. The diameters ranged from 25 to 95 μm and their thicknesses from 16 to 55 μm. Either single cross-sections taken near the half-height of the colony, or *z*-stacks at different depths were taken. Data shown in Fig. 1e and Supplementary Fig. 5 consist of the complete pool of imaged biofilm colonies (*N* = 24), where 19 of them were grown under flow velocity of 0.042 cm/s and the remaining 5 under 0.14 cm/s. Data shown in Fig. 2a, f represent the mean and standard deviations from ten technical replicate measurements at each point of the colony. Data shown in Supplementary Fig. 4 consist of 30 measurements on alginate beads: ten repeated measurements taken at three different points within each bead of different composition.

### Data availability

The data that support the findings of this study are available from the corresponding author upon reasonable request.

## Electronic supplementary material

Supplementary material

Video S.1

Video S.2
