# Supplementary material for: Probing the internal micromechanical properties of Pseudomonas aeruginosa biofilms by Brillouin imaging
Source: NPJ Biofilms Microbiomes. 2017 Sep 8;3:20. doi: 10.1038/s41522-017-0028-z (PMC5591272; doi:10.1038/s41522-017-0028-z)
Supplement: Supplementary file 1 — Supplementary material [file 41522_2017_28_MOESM1_ESM.docx]

**Probing the internal micromechanical properties of *Pseudomonas aeruginosa* biofilms by Brillouin imaging**

A. Karampatzakis, C. Z. Song, L. P. Allsopp, A. Filloux, S. A. Rice, Y. Cohen, T. Wohland and P. Török

**Supplementary material**

**S.1 Brillouin microscope calibration**

Distilled water in a cuvette was used for calibration. A typical spectrum is shown in Figure S.2a, where the Brillouin and Rayleigh peaks are marked with B and R, respectively. The Stokes and anti-Stokes Brillouin peaks are located and define the dispersion axis. The theoretical Brillouin frequency shift of water is 7.04 GHz for a 561 nm laser wavelength. A coefficient that relates frequency shift and peak position in spatial domain *FxP* (frequency per pixel, in GHz) can be calculated as $\frac{FSR-2\times7.04}{B_{2}-B_{1}}$, where FSR is the free spectral range of the VIPA etalon, and $B_{1}$, $B_{2}$ are the positions of the Stokes and anti-Stokes Brillouin peaks along the dispersion axis.

**S.2 Zero-padding**

Zero-padding is commonly used in signal processing to improve speed and precision of the results; it involves appending one or more zeros to the end of a signal. Here, we are using this method to improve signal fitting with Lorentzian curves. The spectrum image can be expressed with an inverse discrete Fourier transformation (IDFT) as:

$$f(m*\delta x)= \sum_{n=0}^{N-1} F(n*\delta u)exp[i2\pi mn/N]$$

where $\delta x$ is the sampling in image space, $\delta u$ is the sampling in Fourier domain, $f(m*\delta x)$ is the spectrum image and $F(n*\delta u)$ is the Discrete Fourier Transform of the spectrum image. It follows that:

$$\delta x=\frac{1}{N*\delta u}$$

Padding *M* zeros in the Fourier Domain increases the number of sampling in the Fourier domain (Figure 3b,c). The sampling in image space therefore reduces according to the relation above and the peak detection accuracy is improved.

**S.3 Control experiments on hydrogel beads**

In order to assess the applicability of the method in bacterial biofilms, we first measured the Brillouin shift in cross-linked Alginate Acid (A. A.) – calcium chloride (CaCl_2_) hydrogel beads (Figure S.3b,c), which are commonly used as model synthetic matrices^1-3^ since alginates are abundant in biofilms and are known to affect their mechanical properties^4^. Figure S.4 shows the Brillouin frequency shifts measured for a number of biologically-relevant alginate concentrations. The values shown are the means of 30 total measurements (10 replicate acquisitions at 3 different points for each bead) and the error bars represent the standard deviations from all measurements for each bead. It can be seen that stiffness was positively correlated to the concentration of the alginate and CaCl_2_. An increase of alginate concentration from 1% to 3% led to an increase in Brillouin shift from 7.02 ± 0.09 GHz to 7.51 ± 0.06 GHz when gelated in a 100 mM CaCl_2_ bath, and from 7.41 ± 0.07 GHz to 7.59 ± 0.05 GHz in 400 mM CaCl_2_. Figure S.2d shows an optical cross-section of a hydrogel bead (100 mM CaCl_2_ and 2% alginate acid). The Brillouin shift measured in the region of interest (ROI) marked by the dashed line was 7.28 ± 0.03 GHz. All measurements were performed with the beads immersed in sterile water.

**Supplementary figures**


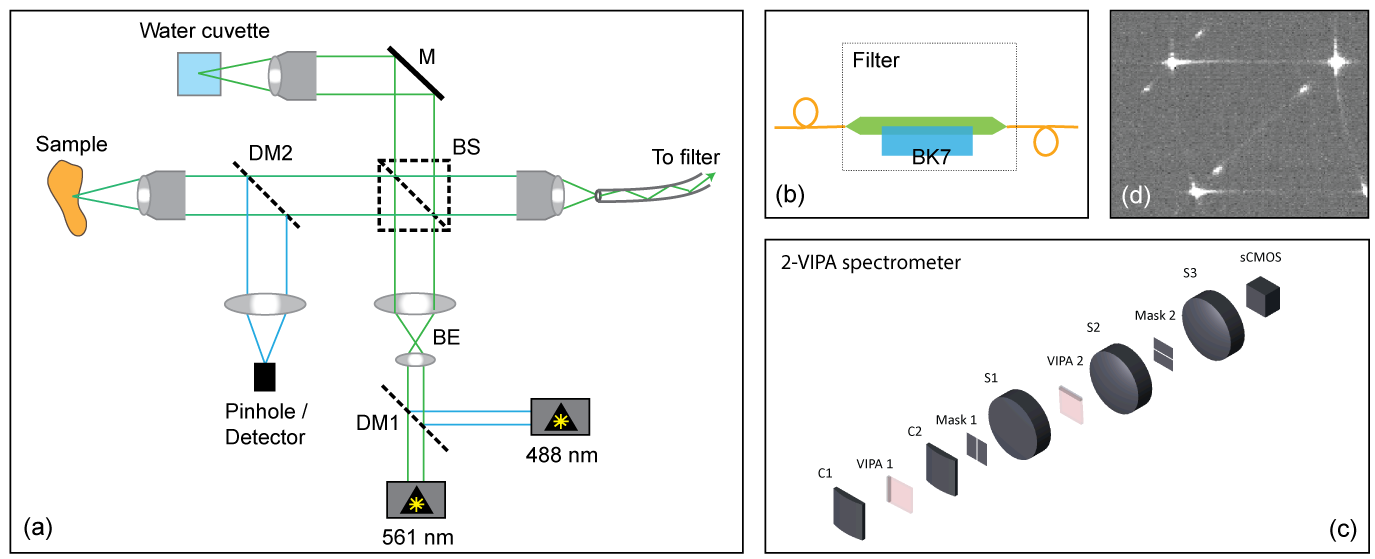


**Figure S.1.** **Imaging setup** (a) Confocal arrangement for Brillouin and fluorescence imaging. DM: Dichroic mirror, BS: beam splitter cube, BE: beam expansion, M: mirror. (b) Schematic of the common-path interferometric filter using BK7 glass slab. (c) Crossed-axis VIPA spectrometer. C1, C2: cylindrical lenses, S1, S2, S3: spherical lenses. (d) Typical spectrum recorded from a biofilm sample using the interferometric filter, 600 ms acquisition time. (a, b, c) drawings are not to scale.


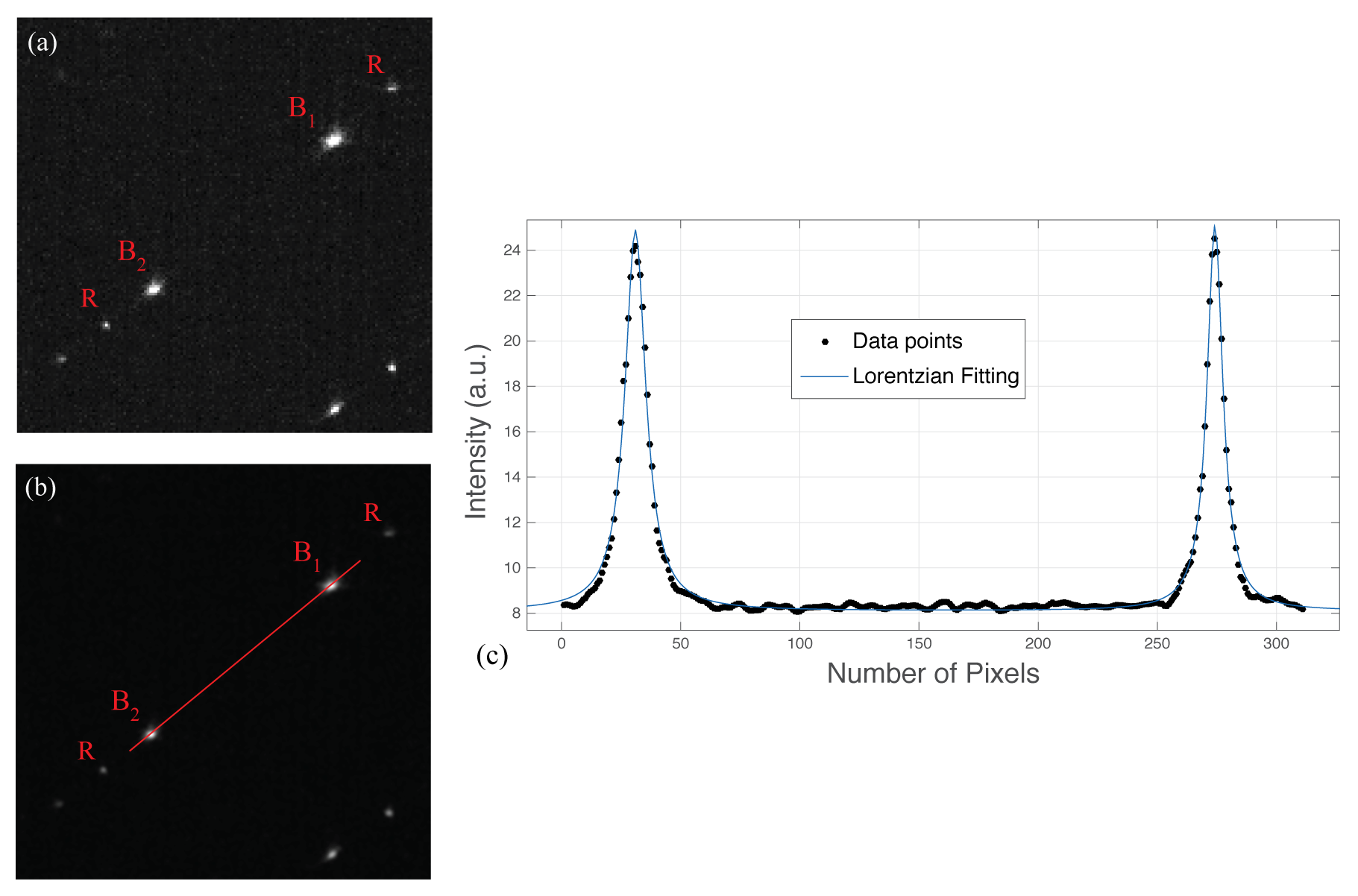


**Figure S.2.** **Spectra of water, used for calibration.** (a) Brillouin spectrum of water, measured by two-stage VIPA spectrometer and interferometer. The Stokes and anti-Stokes Brillouin peaks (B) and Rayleigh peaks (R) are shown. (b) The same image, after zero-padding has been applied. The dispersion axis is marked by the red line. (c) The intensity profile along the dispersion axis is fitted with Lorentzian curves to extract the positions of the Brillouin peaks.

**
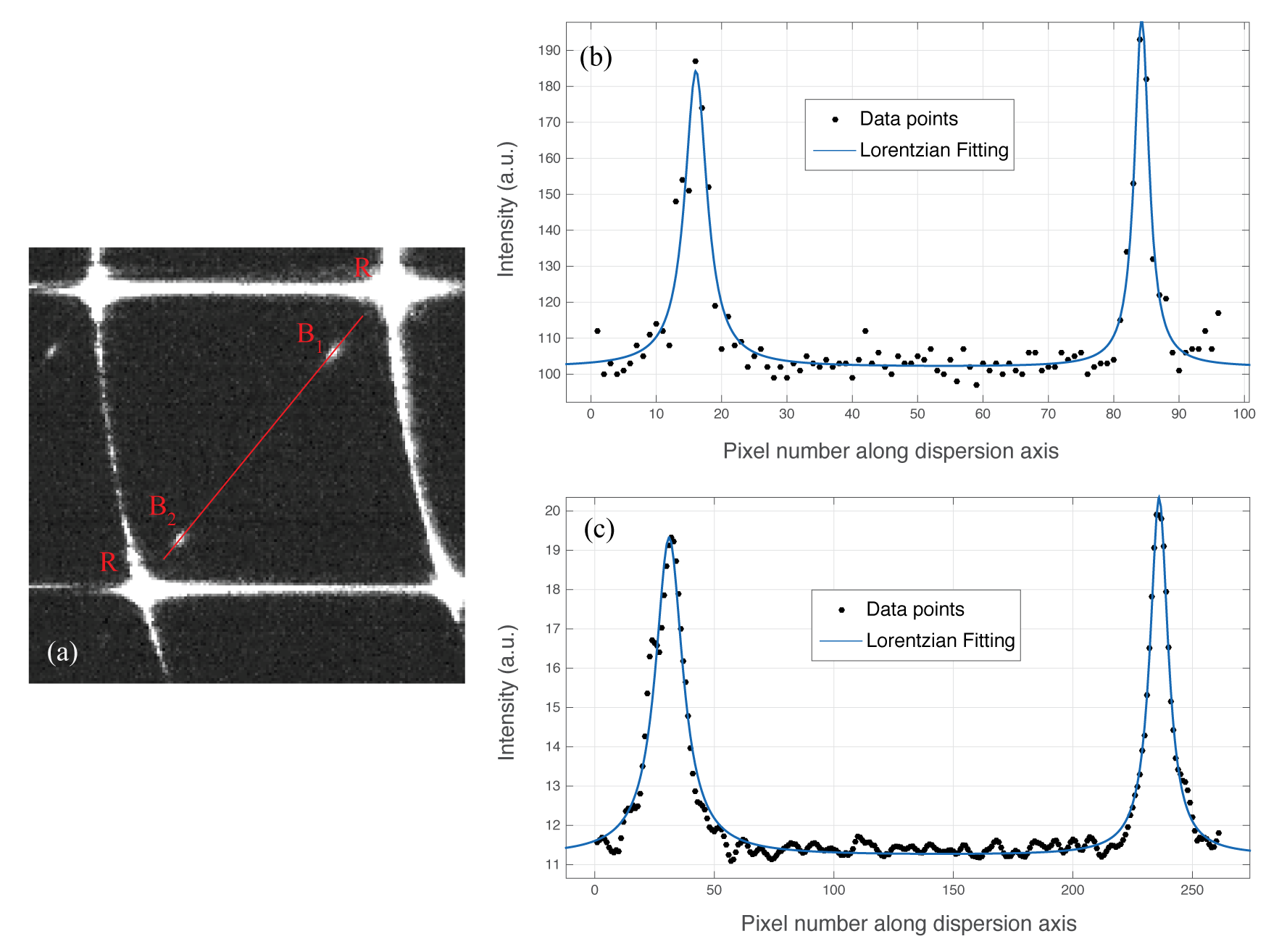
**

**Figure S.3.** **Spectra of a typical biofilm sample.** (a) Brillouin spectrum measured at point inside a biofilm. The Stokes and anti-Stokes Brillouin peaks (B) and Rayleigh peaks (R) are shown. The dispersion axis is marked by the red line. (b, c) Intensity profiles along the dispersion axis, and Lorentzian fits, shown before (b) and after (c) zero-padding.

**
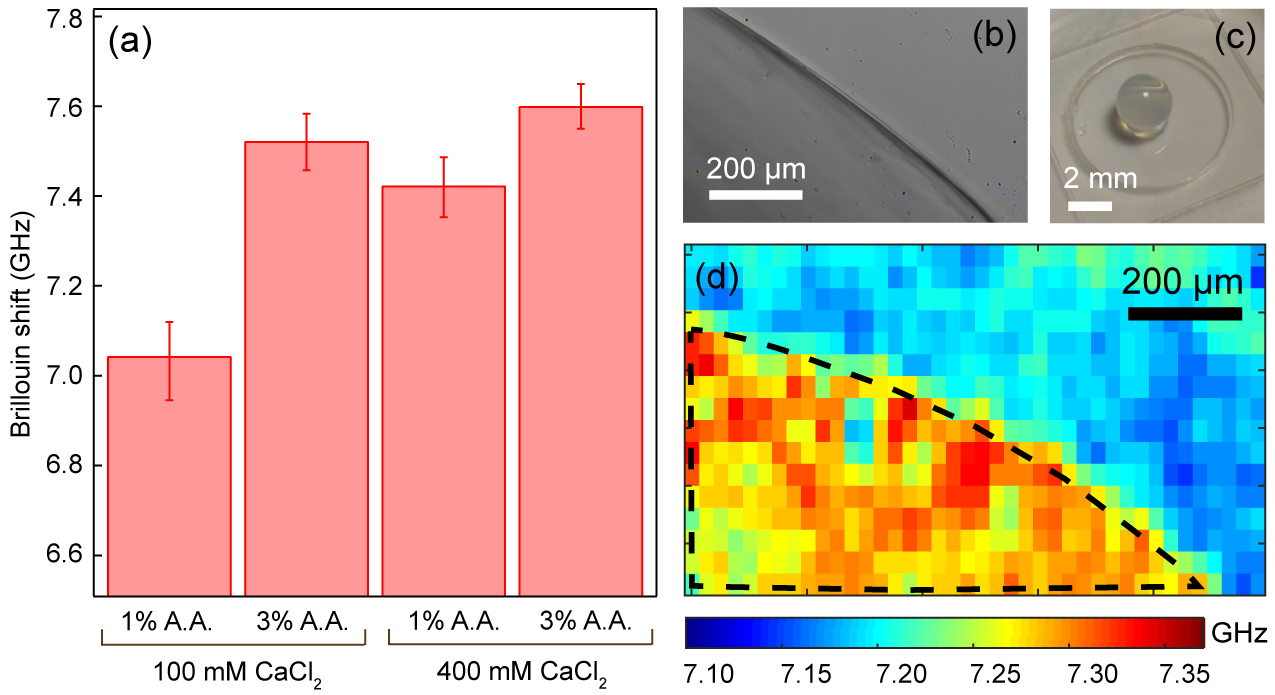
**

**Figure S.4: Mechanical properties of hydrogel beads.** (a) Brillouin shifts measured in crosslinked Alginate Acid (A. A.) – calcium chloride (CaCl_2_) hydrogel beads of different compositions. The error bars denote the standard deviations of 30 measurements: Ten replicate measurements at 3 different points within each bead. (b) Wide field image taken at the interface of a hydrogel bead and water (c) photograph of a typical bead. (d) Brillouin image of a cross section of a hydrogel bead made by crosslinking of 100 mM CaCl_2_ and 2% alginate acid. Note that all measurements were performed with the beads immersed in dH_2_O to avoid dehydration, however, for illustration purposes only, panel (c) shows a bead resting on a petri dish outside water.

**
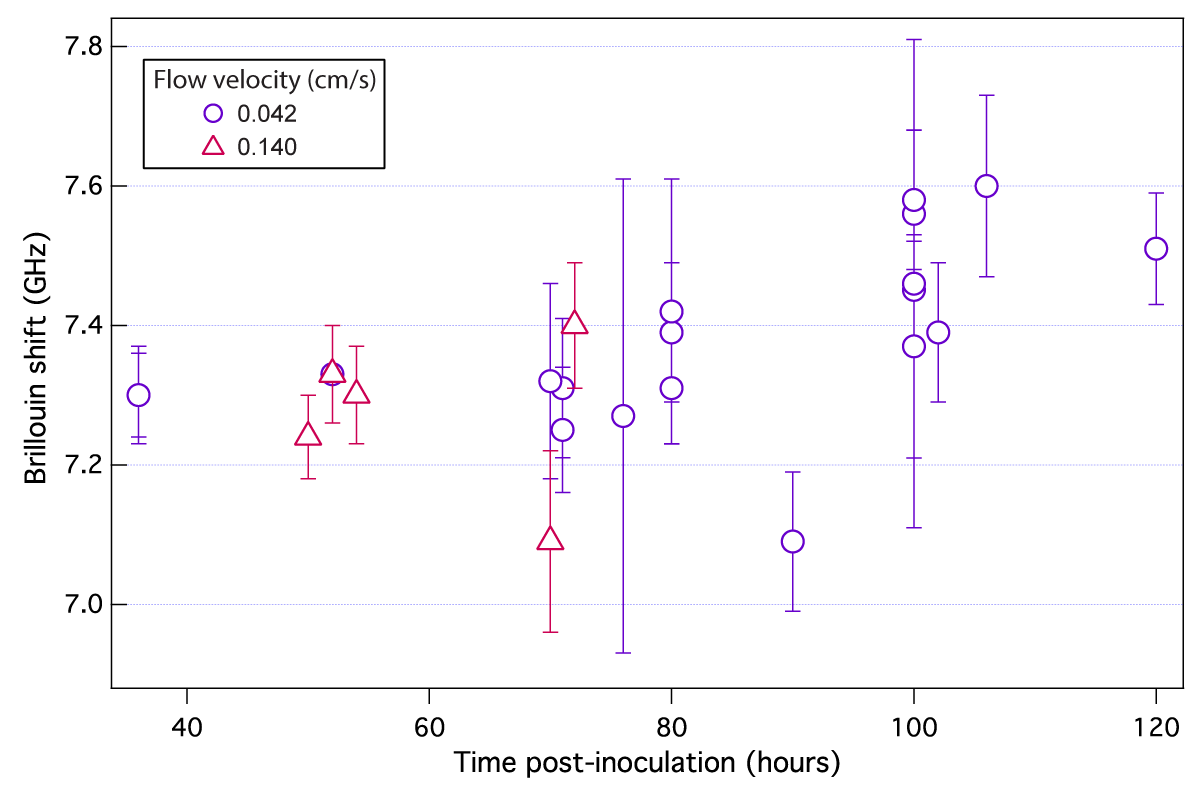
**

**Figure S.5: Mechanical properties of biofilms imaged at different time points, post cell inoculation.** Brillouin shift in colonies of various sizes. Each data point refers to a different colony and denotes the mean and standard deviation from all pixels within the ROIs enclosing the colony. ROIs were drawn by visual inspection of the corresponding widefield images. Biofilms were grown under constant flow velocities of either 0.042 or 0.14 cm/s (circles and triangles, respectively).

**
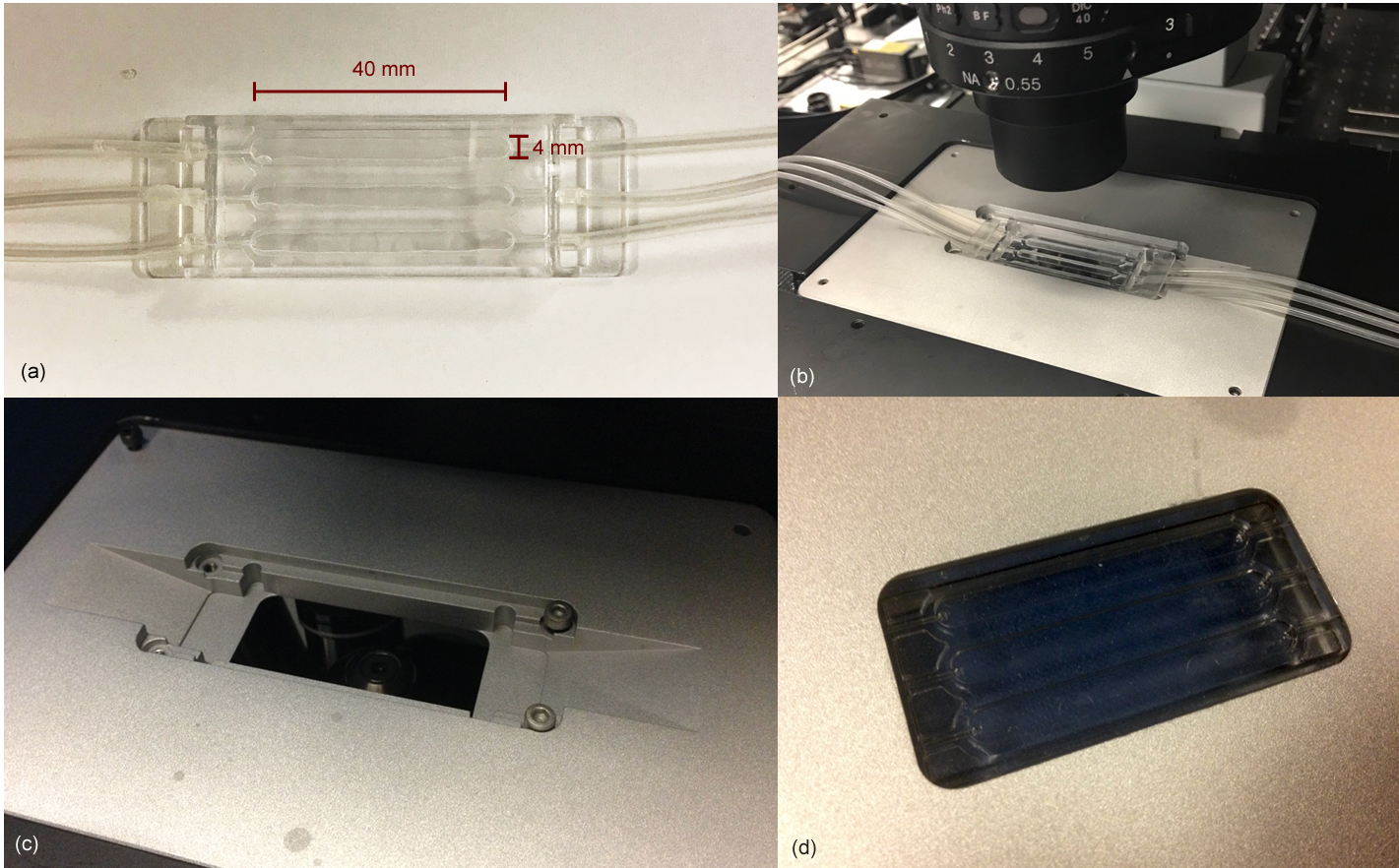
**

**Figure S.6: Flow cell used for imaging the colonies.** (a) The commercial flow cell^5^ used for imaging the colonies as they grow under constant flow. (b) The flow cell on the confocal microscope. (c, d) The stage that was manufactured for the flow cell to be mounted on the confocal microscope.

**Videos**

**Video S.1.** Main panel: Brillouin images taken at different depths inside a typical colony of gfp -tagged *Pseudomonas aeruginosa* (colony thickness = 38 μm, imaged 100 h post inoculation). Inset: Corresponding fluorescence image.

**Video S.2.** Z-stack of widefield image of a mature colony of *Pseudomonas aeruginosa* showing free flowing bacteria around the colony formation and swimming / rotating bacteria inside the colony.

**Supplementary references**

[1] J. A. Rowley, G. Madlambayan, and D. J. Mooney “Alginate hydrogels as synthetic extracellular matrix materials,” Biomaterials **20**, 45-53 (1999).

[2] C. K. Kuo and M. X Ma “Ionically crosslinked alginate hydrogels as scaffolds for tissue engineering: Part 1. Structure, gelation rate and mechanical properties,” Biomaterials **22,** 511–21 (2001).

[3] Y. Zhang, C.K. Ng, Y. Cohen, and B. Cao “Cell growth and protein expression of *Shewanella oneidensis* in biofilms and hydrogel-entrapped cultures,” Molecular BioSystems. **10**, 1035–42 (2014).

[4] O. Orgad, Y. Oren, S. L. Walker, and M. Herzberg “The role of alginate in *Pseudomonas aeruginosa* EPS adherence, viscoelastic properties and cell attachment,” Biofouling **27**(7), 787–98 (2011).

[5] C. Sternberg, and T. Tolker-Nielsen “Growing and Analyzing Biofilms in Flow Cells”. In: R. Coico, T. Kowalik, J. Quarles, B. Stevenson, R. Taylor, editors. “Current Protocols in Microbiology”. New Jersey, JohnWiley. 1B. 2.1–1B. 2.15. 23 (2005).
